# Sex Specific Transcriptional Regulation of Gonadal Steroidogenesis in Teleost Fishes

**DOI:** 10.3389/fendo.2022.820241

**Published:** 2022-02-18

**Authors:** Partigya Sharma, Shriya Purohit, Sachin Kothiyal, Shilpa Negi, Indrashis Bhattacharya

**Affiliations:** Department of Zoology, Hemvati Nandan Bahuguna (HNB) Garhwal University, Pauri Garhwal, India

**Keywords:** transcription factor, steroidogenesis, testosterone, estrogen, cortisol

## Introduction

Fishes are developmentally diverse and economically essential organisms (1). Teleost fishes show remarkable gonadal plasticity by representing both *gonochorism* (one sex at a time) and *hermaphroditism* (more than one sex) (1–3). Gonochoristic teleosts mature as either males or females and remain in such sexual identity throughout adulthood, whereas hermaphroditic species are plastic in terms of sex-reversal signals, i) protogynous (female to male e.g. gilt-head sea bream *Sparus auratus*, bluehead wrasse *Thalassoma bifasciatum*), ii) protandrous (male to female e.g. clown fish *Amphiprion sp, Premnas sp*, Rice filed eel *Monopterus albus* and black porgy *Acanthopagrus schlegeli*) or iii) in both directions for multiple times (e.g. Okinawa goby *Trimma okinawae*, cleaner wrasse *Labroides dimidiatus* and monogamous coral-dwelling gobies *Paragobiodon* and *Gobiodon*) (1, 4). Sexual identity is controlled by social cues and environmental stimuli that operate *via* the cross-talk between hypothalamus–pituitary–gonadal (HPG) and hypothalamus–pituitary– inter-renal (HPI) axes involving neuro-endocrine factors like kisspeptin, dopamine, gonadotropin- releasing hormone (GnRH), gonadotropins (FSH and LH) and gonadal steroids like 17β-estradiol (E_2_) and testosterone (T) (5–7). Gonadal cells initiate the steroidogenic cascade under the influence of FSH and LH by transporting cholesterol from cytosolic lipid droplet to mitochondrial inner membrane *via* Steroidogenic acute regulatory protein (StAR) followed by its rate limited conversion to pregnenolone *via* P450scc enzyme (Cholesterol side chain cleavage enzyme) coded by *Cyp11a1* gene (8). Pregnenolone then gets converted to T through multiple steps prior to the synthesis of bio-active male androgen i.e. 11-Keto-Testosterone (11-KT) or female specific E_2_. The production of E_2_ from T depends on P450- aromatase enzyme coded by *Cyp19a1a* gene in ovaries whereas, T gets converted to 11- hydroxy-androstenedione (11-OHA) by 11β-hydroxylase enzyme coded by *Cyp11b* gene and subsequently 11-OHA acts as a substrate for 11β-hydroxysteroid dehydrogenase (11β-HSD) enzyme coded by *Hsd11b2* gene to produce 11-KT in testes (1, 6–8).

Experimental feminization of XY fries and/or masculinization of XX fries have been successfully achieved by administration of exogenous E_2_ or T for two months respectively (9, 10) despite having a robust genetic mechanism for sex determination (GSD) in medaka *Oryzias latipes* (11, 12). Furthermore, long term depletion of endogenous P450 aromatase by fadrozole in adult teleosts like mekada (13), Nile-tilapia *Oreochromis niloticus* (14) or zebra-fish *Danio rerio* (15) results into functional female to male sex reversal. Consistently, a dramatic shift in plasma sex steroids also has been reported during gonadal trans-differentiation observed in natural sex changing fishes (3). For example, in protogynous species a severe decline in E_2_ leads to ovarian regression followed by a gradual elevation in circulatory 11-KT, whereas in protandrous fishes E_2_ concentration rises with the decline of 11-KT level (2, 16). However, in bidirectional sex change, only E_2_ (not 11-KT) shows such sexual shift in the circulatory pattern (2, 16). Therefore, the critical balance between the bio-conversion rates of T to either E_2_ or 11-KT exclusively directs the sexual fate, reproductive maturation and fertility potential in fishes. The seasonal reproductive cyclicity of fishes is broadly categorized into five stages i) Resting Phase ii) Preparatory/Recrudescence Phase iii) Pre-Spawning/Developing Phase iv) Spawning Active/Capable Phase v) Post-Spawning/Regressing/Spent Phase (17). Intriguingly, a drastic steroidogenic shift occurs in post-vitellogenic (Spawning Phase) ovaries during meiotic maturation of oocytes involving an inhibition of FSH signal leading to the suppression of *Cyp19a1a* promoter and thereby decline in E_2_ production with subsequent LH mediated activation of *20β-Hsd* promoter for the production of maturation inducing steroids [17α,20β-DPs (17α,20β-dihydroxy-4-pregnen-3-one or 17α,20β,21-trihydroxy-4-pregnen-3-one)] (6, 8, 18). Similarly, a dominant upregulation of *Hsd11b2* promoter activity has been reported in testes ensuring the rise in 11-KT level in spawning males. Therefore, a complex interplay among the multiple *cis acting sequences/elements* and respective *trans acting factors* collectively regulate the sex specific differential promoter activities of the genes coding for key steroidogenic enzymes in teleost gonads (8). Although brain, kidney, liver and adipose tissues are other potential sites of steroidogenesis in fishes, we here briefly highlight the critical contribution of major transcription factors regulating gonadal steroidogenic output to determine fish reproduction.

## Glucocorticoid Receptors

Corticosteroid like cortisol produced from adrenal glands acts *via* glucocorticoid receptor (GR) and critically regulates the promoter function of *Cyp19a1a* and *Cyp11c1/Cyp11b* or *Hsd11b2* genes thereby fixing the E_2_: 11-KT concentration (2, 5). Cortisol induced GR blocks the aromatase enzyme in the ovaries of pejerrey *Odontesthes bonariensis* (19) or Japanese flounder *Paralichthys olivaceus* (20) and induces the promoter activity of *Hsd11b2* in the testes of pejerrey (21) or European eel *Anguilla anguilla* (22) in male favourable temperatures.

## Foxl_2_

FOXL_2_ (Forkhead transcription factor 2), member of the winged helix/fork-head group of proteins is known for ovarian differentiation (23). Fox genes like *Foxc1*, *Foxl_2_*, *Foxl_3_* (a germ cell intrinsic transcription factor determinant of sexual fate in medaka) have been shown to determine the ovarian function (23). In ovary, FOXL_2_ suppresses *Dmrt1* and upregulates female programming genes like *Cyp19a1a*, *Rspo1* and *Wnt4/βcatenin* etc and support E_2_ production (23, 24). The co-localizations of FOXL_2_ and P450 aromatase enzyme in the ovaries of adult medaka (25), Nile-tilapia (26–29) and Japanese flounder (30) suggest the critical involvement of FOXL_2_ in transcriptional regulation of *Cyp19a1a* and E_2_ production. In medaka, FOXL_2_ protein is initially detected in the germline stem cells and maintained thereafter throughout the meiotic progression (23). In Nile-tilapia, the promoter region of *Cyp19a1*a possesses the core element ACAAATA from -545 to -538 known for the binding site for FOXL_2_ (27). Over-expression of *Foxl_2_* dominant negative mutant in XX tilapia triggers female to male reversal (27), whereas the loss of *Foxl_2_* in XX tilapia leads to female to male reversal (27, 31–33). In Japanese flounder FOXL_2_ directly activates the *Cyp19a1*a gene transcription by binding to the forkhead- responsive site (30).

## Ad4BP/SF-1

Ad4 Binding Proteins/Steroidogenic Factor1 (AD4BP/SF-1) or Fushi Tarazu factor 1 (FTZ-F1) is an orphan nuclear receptor under subfamily 5 group A member 1 (NR5A1) that gets co-localized in the interstitial cells of pre-vitellogenic ovary and granulosa cells of the vitellogenic follicles along with FOXL_2_ and P450 aromatase in medaka (34, 35) and Nile-tilapia (27). Although in TM3 cell lines and granulosa cells of Nile-tilapia, FOXL_2_ alone can activate the gene transcription of *Cyp19a1a* as both these cells contain endogenous Ad4BP/SF-1, FOXL_2_ alone fails to show such impact on the *Cyp19a1a* promoter in HEK293 cells (27). However, with co-transfection of *Ad4BP/SF1* and *Foxl_2_*, the P450 aromatase promoter gets pronouncedly activated indicating *Foxl_2_* and *Ad4BP/SF1* act synergistically to augment *Cyp19a1a* transcription (36). On the other hand, *Ad4BP/SF-1* binds to two FF1 response elements on the promoter of *Cyp11a1* gene and upregulates its transcription in zebra-fish (37).

## Dax1

DAX1 (Dosage- sensitive sex reversal adrenal hypoplasia congenital critical region on the X chromosome, gene 1), an orphan receptor is a member of the nuclear receptor superfamily (NR0B1). *Dax1* is expressed in adrenal cortex, gonads, ventromedial hypothalamus and pituitary gonadotrophs potentially crucial for testis differentiation (38–40). The expression of *Dax1* is up-regulated by androgen in rainbow trout *Oncorhynchus mykiss* during ovary to testis transition (41). In medaka, DAX1 has only one LXXLL- related motif in N-terminal and is involved in repressing E_2_ synthesis in ovarian follicles (36). In medaka co-transfection of *Dax1* along with *AD4BP/SF-1* and *Foxl_2_* in a dose dependent manner leads to a significant decline in the activity of *Cyp19a1a* promoter indicating that DAX1 negatively regulates *Cyp19a1a* expression by suppressing Ad4BP/SF1 and FOXL*_2_* proteins in ovarian follicles (36).

## Dmy/Dmrt 1

In metazoans, double-sex and mab-3 related transcription factor 1 (*Dmrt1*) is the critical inducer of testicular differentiation (42). Testes restricted expression patterns of *Dmrt1* have been found in medaka, Nile-tilapia, Olive flounder, Rainbow trout African catfish *Clarias gariepinus*, rare minnow *Gobiocypris rarus*, lake sturgeon *Acipenser fulvescens*, Atlantic cod *Gadus morhua*, pejerrey,shovelnose sturgeon *Scaphirhynchus platorynchus* and southern catfish *Silurus meridionals* (42). DMRT1 either alone or in synergy with DAX1 represses the female programming genes like *Cyp19a1a*, *Rspo1*, *Figla*, *Gdf9* and *Wnt4/β catenin* and augments the transcription of male specific genes like *Gsdf*, *Cyp11c1*, *Sox9/3*, *Amh* etc in testes (42). The exposure of E_2_ downregulates *Dmrt1* transcription in medaka (43), African catfish (44), Nile-tilapia (28), rare minnow (45), pejerrey (46), zebra-fish (47) and rainbow trout (48). DMRT1 directly suppresses *Cyp19a1a* promoter activity in medaka (36) and in Nile-tilapia (28). In XX tilapia, overexpression of *Dmrt1* leads to downregulation of *Cyp19a1a* expression and E_2_ production (28). However, knock-down of *Dmrt1* in XY tilapia (32) and mutation of *Dmrt1* in *Cynoglossusse milaevis* (49) resulted in increased *Foxl_2_* and *Cyp19a1a* expression without any male to female sex reversal. On the other hand, the loss of *Dmrt1* leads to an elevated expression of *Foxl_2_* in zebra-fish (50) whereas an augmentation in both *Foxl_2_* and *Cyp19a1a* in medaka (51). The duplicated copy of *Dmrt1a* on the Y chromosome *Dmy/Dmrt1bY* (DM domain gene on the Y chromosome/doublesex and mab-3 related transcription factor 1b on the Y chromosome) acts as a master of male sex in medaka (12, 52). However, *Dmy* downregulates itself by binding to the conserved cis-regulatory elements like *Izanagi*, within its promoter (42). In differentiating testes of medaka, *Dmy* downregulates the hedgehog pathway by suppressing its receptor *Pitch-2* and upregulating its antagonist *Hhip* (42).

## Sox Proteins

The Sry related HMG box (*Sox*) gene(s) encode variety of transcription factor(s) critical for gonadal morphogenesis (5). Two paralogous forms of *Sox9*, namely *Sox9a* and *Sox9b* are reported in medaka and zebra-fish without any such sexual dimorphic expression pattern (53). In Indian rice-fish *Oryzias dancena*, *Sox3* gene has been shown critical for sex determination by up-regulating expression of *Gsdf* (Gonadal soma-derived factor) (54). The expressions of *Sox3* and *Hsd11b2* are found to be associated with the initiation and progression of spermatogenesis in male catfish testis (8). Precisely, SOX3 binds to *Hsd11b2* gene promote and transactivates its transcription in males (8). During development of zebra-fish, the transcription factor SOX5, directly downregulates the *Dmrt1* transcription (42) whereas SOX5 downregulates *Cyp19a1a* transcription in the red spotted grouper *Epinephelus akaara* (55).

## Other Transcription Factors

Wilm’s tumor 1 (WT1) is a key transcription factor having critical role in mammalian gonadal morphogenesis (5). In Indian catfish *Clarias batrachus*, WT1 has been shown to upregulate the promoter activity of *Hsd11b2* (56). E_2_ bound Estrogen Receptor (coded by *Esr1* gene) acts as a potential transcriptional inducer for *Cyp19a1a* in zebra-fish (57, 58). Finally, activated cAMP response element binding protein (CREB-P; phosphorylated at Ser-133 residue) differentially regulate the transcriptional control of *Cyp19a1a* and *20β-Hsd* genes as found in ovaries of Nile-tilapia, rainbow trout and catfishes (8, 18). In pre-spawning vitellogenic ovaries, CREB-P upregulates the transcription of *Cyp19a1a* in synergy with Ad4BP/SF-1 and FOXL_2_ under the influence of FSH. However, in post-vitellogenic spawning ovaries such dominant transcription of *Cyp19a1a* gets downregulated due to selective inhibition of FSH signal followed by LH induced upregulation *20β-Hsd* transcription by CREB-P alone synthesizing maturation inducing steroids (17α,20β-DPs) critical for the meiotic progression of developing oocytes (8, 18).

## Conclusion

**Figure 1:**
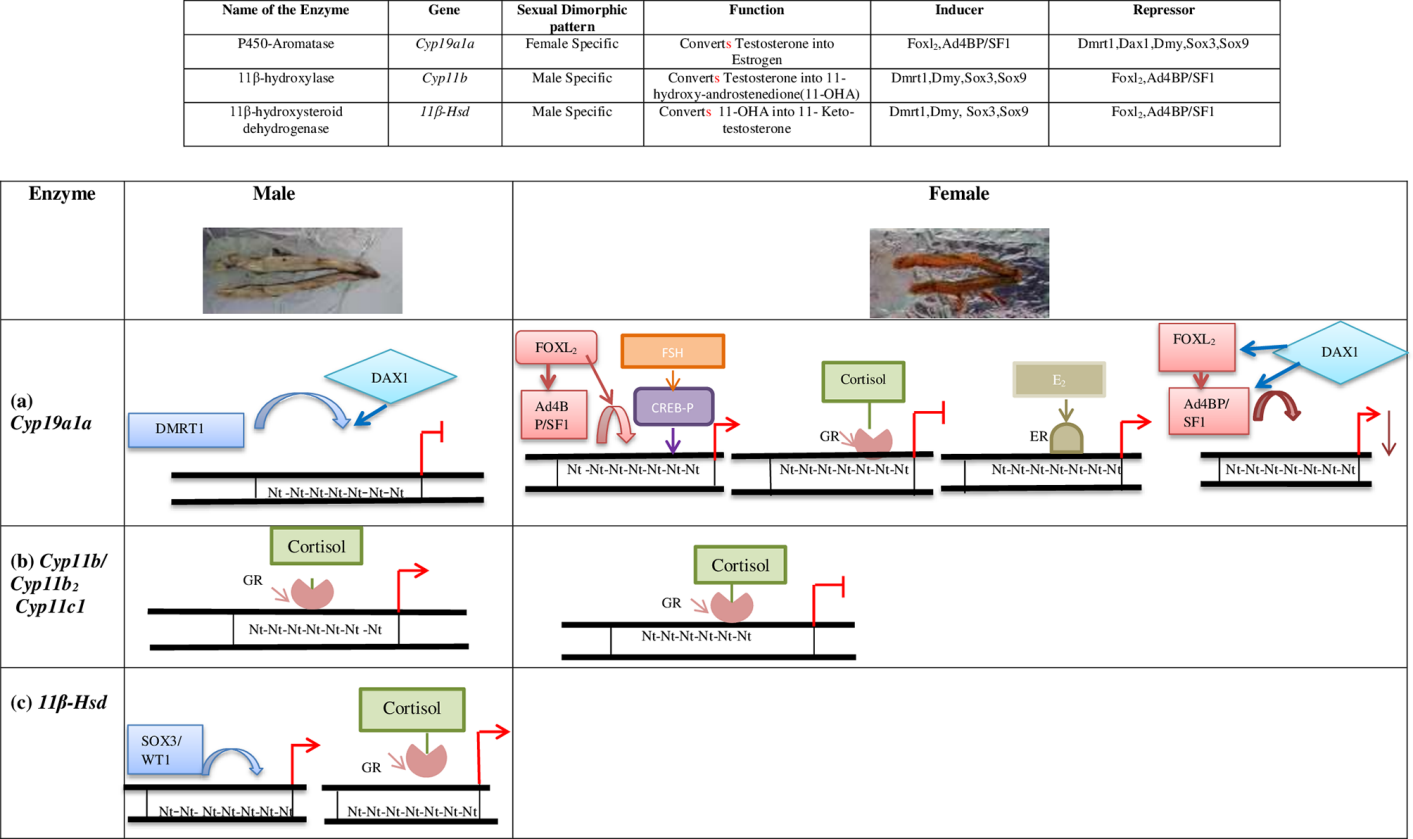
Sex specific regulation of gonadal steroidogenesis by transcription factors. Images of testis and ovary are representatives of a typical fresh water teleost (*Schizothorax plagiostomus, Order: Cypriniformes, Family: Cyprinidae*). **(A)** Regulation of *Cyp19a1a* promoter: In testis, DMRT1 alone or in synergy with DAX1 represses the *Cyp19a1a* transcription. In ovaries, FOXL_2_ either alone or along with Ad4BP/SF-1 activates *Cyp19a1a* transcription. CREB-P upregulates the transcription of *Cyp19a1a* in synergy with FOXL_2_ and Ad4BP/SF1 under the influence of FSH. Cortisol induced Glucocorticoid Receptor (GR) blocks the aromatase enzyme. Estrogen bound Estrogen Receptor (ER) acts as a potential inducer of *Cyp19a1a* transcription. DAX1 negatively regulates the *Cyp19a1a* expression by suppressing Ad4BP/SF-1 and FOXL_2_ **(B)** Regulation of *Cyp11b/Cyp11b_2_/Cyp11c1* promoters: Cortisol induced GR activates the transcription of *Cyp11b/Cyp11b_2_/Cyp11c1* in males. **(C)** Regulation of *11β- Hsd* promoter: In males, SOX3/WT1 binds to *11β-Hsd* and promoters its transcription and Cortisol induced GR activates the transcription of *11β-Hsd.* 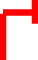: Repression 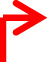: Activation 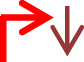: Decline in activity, Nt, Nucleotide sequence.

In summary, molecular techniques like Electrophoretic mobility shift assay (EMSA) or Chromatin immuno-precipitation (ChIP) assay investigating DNA-protein interactions have revealed the differential promoter activities of the key steroidogenic genes like *Cyp19a1a*, *Cyp11b* and *Hsd11b2* by various transcription factors to regulate the turnover rate of T for fixing the E_2_: 11-KT concentration in fish gonads (8). For example, in males, DMRT1 directly suppresses *Cyp19a1a* transcription, while DAX1 does the same *via* inhibiting FOXL_2_ and Ad4BP/SF-1 leading to the testicular differentiation. Conversely, FOXL_2_ and Ad4BP/SF-1 augment the expression of *Cyp19a1a* ensuring E_2_ production and promote the ovarian function. **Figure 1** schematically represents the complex antagonistic genetic network that regulates the ovarian estrogenic or testicular androgenic milieu in teleost gonads. **Supplementary Table 1** describes the critical role(s) of major transcription factors identified till date in different teleost species in a chronological order directing gonadal development and function. **Supplementary Table 2** summarizes the effect of various environmental parameters on fish gonadal development and steroidogenesis.

## Future Directions

Notably there is a potential scope for the commercial application of genetically engineered fishes having mutant transcription factors by advanced genome editing technologies like TALEN/CRISPER-CAS9 Transgenesis/RNAi mediated silencing etc. These gain in function (by transgenic over-expression/integration) or loss of function (by knock-out/down) models can be successfully implemented in aquaculture or fishery industry to increase the productivity/yield by manipulating fertility of fishes. However more studies are required in non-model teleost species by employing advanced high throughput next generation sequencing with multi-omics approach to generate gonadal transcriptomic resource datasets. This may further help identify the involvement of new putative factor(s) like chromatin remodelling complexes, DNA-methyl-transferases (DNMTs) and novel micro-RNAs or long-non-coding RNAs which can potentially regulate the sex specific transcriptional switch in gonadal steroidogenesis (59).

## Author Contributions

IB conceived the idea. PS wrote the first draft of the Text, Figure, and Table with support from SP, SK and SN. IB generated the final form of the manuscript. All authors contributed to the article and approved the submitted version.

## Funidng

IB acknowledges the financial support from University Grants Commission (F.30104/2015BSR) and Department of Science and Technology (ECR/2018/000868). The funder was not involved in the study design, collection, analysis, interpretation of data, the writing of this article or the decision to submit it for publication.

## Conflict of Interest

The authors declare that the research was conducted in the absence of any commercial or financial relationships that could be construed as a potential conflict of interest.

## Publisher’s Note

All claims expressed in this article are solely those of the authors and do not necessarily represent those of their affiliated organizations, or those of the publisher, the editors and the reviewers. Any product that may be evaluated in this article, or claim that may be made by its manufacturer, is not guaranteed or endorsed by the publisher.
