# Supplementary material for: Sex Specific Transcriptional Regulation of Gonadal Steroidogenesis in Teleost Fishes
Source: Front Endocrinol (Lausanne). 2022 Feb 18;13:820241. doi: 10.3389/fendo.2022.820241 (PMC8894591; doi:10.3389/fendo.2022.820241)
Supplement: Supplementary file 1 [file Table_1.docx]

**Supplementary Table 1 Summarizing critical role of Transcription factors regulating Gonadal development and functions**

| **A. *Dmrt/Dmrt1Y/Dmy -*transcription factor with a conserved DNA-binding motif known as the *doublesex and mab-3-realted* domain. This domain is a non-canonical cysteine-rich DNA binding motif with two intertwined finger structures chelating one zinc ion that binds to the minor groove of DNA** | | | | | |
| --- | --- | --- | --- | --- | --- |
| **Year** | | **Species** | | **Function** | **Reference** |
| 2002 | | *Oryzias latipes* | | *Dmrt1Y* determines the male sex gene in medaka. | Nanda, I., Kondo, M., Hornung, U., Asakawa, S., Winkler, C., Shimizu, A., Shan, Z., Haaf, T., Shimizu, N., Shima, A., Schmid, M., & Schartl, M. (2002). A duplicated copy of DMRT1 in the sex-determining region of the Y chromosome of the medaka, Oryzias latipes. *Proceedings of the National Academy of Sciences of the United States of America*, *99*(18), 11778–11783.  doi:10.1073/pnas.182314699. |
| 2002 | | *Oryzias latipes* | | *DMY* is required for testicular development and is a prime candidate for the medaka sex determining gene in medaka. | Matsuda, M., Nagahama, Y., Shinomiya, A., Sato, T., Matsuda, C., Kobayashi, T., Morrey, C. E., Shibata, N., Asakawa, S., Shimizu, N., Hori, H., Hamaguchi, S., & Sakaizumi, M. (2002). DMY is a Y-specific DM-domain gene required for male development in the medaka fish. *Nature*, *417*(6888), 559–563. doi:10.1038/nature751 |
| 2004 | | *Oryzias latipes* | | *Dmrt1* regulates spermatogonial differentiation. During early gonadal differentiation of XY individuals, *DMY* regulates Primordial Germ Cell proliferation and differentiation sex-specifically. | Kobayashi, T., Matsuda, M., Kajiura-Kobayashi, H., Suzuki, A., Saito, N., Nakamoto, M., Shibata, N., & Nagahama, Y. (2004). Two DM domain genes, DMY and DMRT1, involved in testicular differentiation and development in the medaka, Oryzias latipes. *Developmental dynamics : an official publication of the American Association of Anatomists*, *231*(3), 518–526.  doi:10.1002/dvdy.20158. |
| 2004 | | *Danio rerio* | | *Dmrt5* have potential role in gonadal development and may have contributed to the functional endocrine axis. | Guo, Y., Li, Q., Gao, S., Zhou, X., He, Y., Shang, X., Cheng, H., & Zhou, R. (2004). Molecular cloning, characterization, and expression in brain and gonad of Dmrt5 of zebrafish. *Biochemical and biophysical research communications*, *324*(2), 569–575. doi:10.1016/j.bbrc.2004.09.085 |
| 2005 | | *Danio rerio* | | *Dmrt1* gene not only associated with testis development, but also have important role in Ovary differentiation. | Guo, Y., Cheng, H., Huang, X., Gao, S., Yu, H., & Zhou, R. (2005). Gene structure, multiple alternative splicing, and expression in gonads of zebrafish Dmrt1. *Biochemical and biophysical research communications*, *330*(3), 950–957. doi:10.1016/j.bbrc.2005.03.066 |
| 2005 | | *Danio rerio* | | SOX5 bound to the promoter of *Dmrt1* and inhibits its expression. This antagonistic partnership between *Dmrt1* and *Sox5* suggests a potential transcriptional regulatory mechanism for *Dmrt1* in early embryogenesis. | Gao, S., Zhang, T., Zhou, X., Zhao, Y., Li, Q., Guo, Y., Cheng, H., & Zhou, R. (2005). Molecular cloning, expression of Sox5 and its down-regulation of Dmrt1 transcription in zebrafish. *Journal of experimental zoology. Part B, Molecular and developmental evolution*, *304*(5), 476–483.  doi:10.1002/jez.b.21053 |
| 2006 | | *Takifugu rubripes* | | *Dmrt1* involved in gonadal development rather than sex differentiation and its expression correlates with the proliferation of spermatogonia. | Yamaguchi, A., Lee, K. H., Fujimoto, H., Kadomura, K., Yasumoto, S., & Matsuyama, M. (2006). Expression of the DMRT gene and its roles in early gonadal development of the Japanese pufferfish Takifugu rubripes. *Comparative biochemistry and physiology. Part D, Genomics & proteomics*, *1*(1), 59–68. doi:10.1016/j.cbd.2005.08.003 |
| 2006 | | *Xiphophorus maculatus* | | *Dmrt1* expression is detected in both spermatogonia and Sertoli cells in Platyfish. | Veith, A. M., Schäfer, M., Klüver, N., Schmidt, C., Schultheis, C., Schartl, M., Winkler, C., & Volff, J. N. (2006). Tissue-specific expression of dmrt genes in embryos and adults of the platyfish Xiphophorus maculatus. *Zebrafish*, *3*(3), 325–337. doi:10.1089/zeb.2006.3.325 |
| 2007 | | *Oryzias latipes* | | *Dmy* is able to induce testis differentiation and subsequent male development in XX ( genetically female) medaka. | Matsuda, M., Shinomiya, A., Kinoshita, M., Suzuki, A., Kobayashi, T., Paul-Prasanth, B., Lau, E. L., Hamaguchi, S., Sakaizumi, M., & Nagahama, Y. (2007). DMY gene induces male development in genetically female (XX) medaka fish. *Proceedings of the National Academy of Sciences of the United States of America*, *104*(10), 3865–3870. doi:10.1073/pnas.0611707104 |
| 2008 | | *Oreochromis niloticus* | | *Dmrt1* is a superior testicular differentiation marker. | Kobayashi, T., Kajiura-Kobayashi, H., Guan, G., & Nagahama, Y. (2008). Sexual dimorphic expression of DMRT1 and Sox9a during gonadal differentiation and hormone-induced sex reversal in the teleost fish Nile tilapia (Oreochromis niloticus). *Developmental dynamics : an official publication of the American Association of Anatomists*, *237*(1), 297–306.  doi:10.1002/dvdy.21409 |
| 2008 | | *Danio rerio* | | *Dmrt3* may function in the nucleus as a effective transcription factor to exert potential roles in the development of the olfactory placode, the neural tube and germ cells. | Li, Q., Zhou, X., Guo, Y., Shang, X., Chen, H., Lu, H., Cheng, H., & Zhou, R. (2008). Nuclear localization, DNA binding and restricted expression in neural and germ cells of zebrafish Dmrt3. *Biology of the cell*, *100*(8), 453–463.  doi:10.1042/BC20070114 |
| 2010 | | *Oryzias latipes* | | In genetic females of medaka, introduction of *Dmrt1bY* transgene leads testis development. | Otake, H., Masuyama, H., Mashima, Y., Shinomiya, A., Myosho, T., Nagahama, Y., Matsuda, M., Hamaguchi, S., & Sakaizumi, M. (2010). Heritable artificial sex chromosomes in the medaka, Oryzias latipes. *Heredity*, *105*(3), 247–256.  doi:10.1038/hdy.2009.174 |
| 2009 | | *Clarias gariepinus* | | DMRT1 protein is present in spermatogonia and spermatocytes, which indicates plausible role in spermatogenesis. The potential role for DMRT1 in testicular differentiation is evident from its stage dependent elevated expression in developing testis. | Raghuveer, K., & Senthilkumaran, B. (2009). Identification of multiple dmrt1s in catfish: localization, dimorphic expression pattern, changes during testicular cycle and after methyltestosterone treatment. *Journal of molecular endocrinology*, *42*(5), 437–448. doi:10.1677/JME-09-0011 |
| 2009 | | *Paralichthys olivaceus* | | There is a possible role of *Dmrt4* in the development of the gonads, nervous system and sense organs. | Wen, A., You, F., Tan, X., Sun, P., Ni, J., Zhang, Y., Xu, D., Wu, Z., Xu, Y., & Zhang, P. (2009). Expression pattern of dmrt4 from olive flounder (Paralichthys olivaceus) in adult gonads and during embryogenesis. *Fish physiology and biochemistry*, *35*(3), 421–433.  doi:10.1007/s10695-008-9267-5 |
| 2010 | | *Gadus morhua* | | Sexually dimorphic expression of *Dmrt1* was documented by quantitative PCR with the highest mRNA levels in immature males corresponding to the start of spermatogenesis. | Johnsen, H., Seppola, M., Torgersen, J. S., Delghandi, M., & Andersen, Ø. (2010). Sexually dimorphic expression of dmrt1 in immature and mature Atlantic cod (Gadus morhua L.). *Comparative biochemistry and physiology. Part B, Biochemistry & molecular biology*, *156*(3), 197–205. doi:10.1016/j.cbpb.2010.03.009 |
| 2010 | | *Oreochromis aureus* | | In gonadal development, *Dmrt4* gene have potentially important roles, and may have contributed to the functional endocrine axis. | Cao, J., Chen, J., Wu, T., Gan, X., & Luo, Y. (2010). Molecular cloning and sexually dimorphic expression of DMRT4 gene in Oreochromis aureus. *Molecular biology reports*, *37*(6), 2781–2788.  doi:10.1007/s11033-009-9820-z |
| 2010 | | *Oreochromis niloticus* | | *In vitro* analysis using luciferase assays revealed that *Dmrt1* repressed basal as well as *Ad4BP/SF-1* activated *Cyp19a1a* transcription in HEK293 Cells. *Dmrt1* suppresses the female pathway by repressing aromatase gene transcription and estrogen production in the gonads of tilapia and possibly other vertebrates. | Wang, D. S., Zhou, L. Y., Kobayashi, T., Matsuda, M., Shibata, Y., Sakai, F., & Nagahama, Y. (2010). Doublesex- and Mab-3-related transcription factor-1 repression of aromatase transcription, a possible mechanism favoring the male pathway in tilapia. *Endocrinology*, *151*(3), 1331–1340. doi:10.1210/en.2009-0999 |
| 2012 | | *Acanthopagrus schlegeli* | | *Dmrt1* plays a key role in initial testis differentiation and in later maintenance of male development. | Wu, G. C., Chiu, P. C., Lin, C. J., Lyu, Y. S., Lan, D. S., & Chang, C. F. (2012). Testicular dmrt1 is involved in the sexual fate of the ovotestis in the protandrous black porgy. *Biology of reproduction*, *86*(2), 41. doi:10.1095/biolreprod.111.095695 |
| 2012 | | *Gobiocypris rarus* | | *Dmrt1* plays critical role in sex determination and sex differentiation. | Cao, M., Duan, J., Cheng, N., Zhong, X., Wang, Z., Hu, W., & Zhao, H. (2012). Sexually dimorphic and ontogenetic expression of dmrt1, cyp19a1a and cyp19a1b in Gobiocypris rarus. *Comparative biochemistry and physiology. Part A, Molecular & integrative physiology*, *162*(4), 303–309. doi:10.1016/j.cbpa.2012.03.021 |
| 2012 | | *Oryzias latipes* | | *Dmrt1* is essential to maintain testis differentiation. XY medaka having mutated *Dmrt1* l shows trans differentiation of testis to Ovary, phenotypic rescue is possible by transgenic *Dmrt1* integration. | Masuyama, H., Yamada, M., Kamei, Y., Fujiwara-Ishikawa, T., Todo, T., Nagahama, Y., & Matsuda, M. (2012). Dmrt1 mutation causes a male-to-female sex reversal after the sex determination by Dmy in the medaka. *Chromosome research*, *20*(1), 163-176.  doi:10.1007/s10577-011-9264-x |
| 2013 | | *Oreochromis niloticus* | | *Dmrt1* and/or *Amh* may be the modulator(s) of the down-regulation of *Foxl_2_* and/or *Cyp19a1a.* | Poonlaphdecha, S., Pepey, E., Canonne, M., de Verdal, H., Baroiller, J. F., & D'Cotta, H. (2013). Temperature induced-masculinisation in the Nile tilapia causes rapid up-regulation of both dmrt1 and amh expressions. *General and comparative endocrinology*, *193*, 234–242. doi:10.1016/j.ygcen.2013.06.007 |
| 2014 | | *Oreochromis niloticus* | | The *Dmrt6* gene is highly expressed in spermatocytes and is involved in spermatogenesis in tilapia and reduce *Cyp11b2* and low level of serum of 11-KT. | Zhang, X., Wang, H., Li, M., Cheng, Y., Jiang, D., Sun, L., Tao, W., Zhou, L., Wang, Z., & Wang, D. (2014). Isolation of doublesex- and mab-3-related transcription factor 6 and its involvement in spermatogenesis in tilapia. *Biology of reproduction*, *91*(6), 136. doi:10.1095/biolreprod.114.121418 |
| 2014 | | *Sebastes schlegeli* | | *Dmrt1* may have an important role in the differentiation of both the testis and the ovary of *S. schlegeli*. | Ma, L., Wang, W., Yang, X., Jiang, J., Song, H., Jiang, H., Zhang, Q., & Qi, J. (2014). Characterization of the Dmrt1 gene in the black rockfish Sebastes schlegeli revealed a remarkable sex-dimorphic expression. *Fish physiology and biochemistry*, *40*(4), 1263–1274. doi:10.1007/s10695-014-9921-z |
| 2014 | | Gibel carp | | *Dmrt1* is related to testis differentiation and spermatogenesis. | Li, X. Y., Li, Z., Zhang, X. J., Zhou, L., & Gui, J. F. (2014). Expression characterization of testicular DMRT1 in both Sertoli cells and spermatogenic cells of polyploid gibel carp. *Gene*, *548*(1), 119–125. doi:10.1016/j.gene.2014.07.031 |
| 2015 | | *Astyanax altiparanae* | | After spermiation, *Dmrt1*and *Sox9* are down-regulated indicating a role of these genes in spermatogenesis in male reproductive cycle. | Adolfi, M. C., Carreira, A. C., Jesus, L. W., Bogerd, J., Funes, R. M., Schartl, M., Sogayar, M. C., & Borella, M. I. (2015). Molecular cloning and expression analysis of dmrt1 and sox9 during gonad development and male reproductive cycle in the lambari fish, Astyanax altiparanae. *Reproductive biology and endocrinology : RB&E*, *13*, 2.  doi:10.1186/1477-7827-13-2 |
| 2016 | | *Acipenser sinensis* | | *Dmrt1* appears to be a highly specific sex differentiation gene for testis development and spermatogenesis. | Leng, X. Q., Du, H. J., Li, C. J., & Cao, H. (2016). Molecular characterization and expression pattern of dmrt1 in the immature Chinese sturgeon Acipenser sinensis. *Journal of fish biology*, *88*(2), 567–579. doi:10.1111/jfb.12852 |
| 2017 | | *Danio rerio* | | *Dmrt1* is necessary for testis development. | Webster, K. A., Schach, U., Ordaz, A., Steinfeld, J. S., Draper, B. W., & Siegfried, K. R. (2017). Dmrt1 is necessary for male sexual development in zebrafish. *Developmental biology*, *422*(1), 33–46. doi:10.1016/j.ydbio.2016.12.008 |
| 2017 | | *Cynoglossus semilaevis* | | *Dmrt1* is male sex determining gene. | Cui, Z., Liu, Y., Wang, W., Wang, Q., Zhang, N., Lin, F., Wang, N., Shao, C., Dong, Z., Li, Y., Yang, Y., Hu, M., Li, H., Gao, F., Wei, Z., Meng, L., Liu, Y., Wei, M., Zhu, Y., Guo, H., … Chen, S. (2017). Genome editing reveals dmrt1 as an essential male sex-determining gene in Chinese tongue sole (Cynoglossus semilaevis). *Scientific reports*, *7*, 42213. doi:10.1038/srep42213 |
| 2017 | | *Danio rerio* | | *Dmrt1* might be required for the maintenance, self-renewal and differentiation of male germ cells. | Lin, Q., Mei, J., Li, Z., Zhang, X., Zhou, L., & Gui, J. F. (2017). Distinct and Cooperative Roles of *amh* and *dmrt1* in Self-Renewal and Differentiation of Male Germ Cells in Zebrafish. *Genetics*, *207*(3), 1007–1022. doi:10.1534/genetics.117.300274 |
| 2019 | | *Oreochromis niloticus* | | By directly binding to specific site within the *Sox30* promoter, *Dmrt1* positively regulates its transcription. | Tang, Y., Li, X., Xiao, H., Li, M., Li, Y., Wang, D., & Wei, L. (2019). Transcription of the *Sox30* Gene Is Positively Regulated by Dmrt1 in Nile Tilapia. *International journal of molecular sciences*, *20*(21), 5487.  doi:10.3390/ijms20215487 |
| 2019 | | *Micropterus salmoides* | | *Dmrt1* plays an important role in sex determination and differentiation. | Yan, N., Hu, J., Li, J., Dong, J., Sun, C., & Ye, X. (2019). Genomic organization and sexually dimorphic expression of the Dmrt1 gene in largemouth bass (Micropterus salmoides). *Comparative biochemistry and physiology. Part B, Biochemistry & molecular biology*, *234*, 68–77.  doi:10.1016/j.cbpb.2019.05.005 |
| 2019 | | *Oreochromis niloticus* | | Male sex-differentiation factor *Dmrt1* positively regulates the transcription of the Nile tilapia *Sox9b*. | Wei, L., Li, X., Li, M., Tang, Y., Wei, J., & Wang, D. (2019). Dmrt1 directly regulates the transcription of the testis-biased Sox9b gene in Nile tilapia (Oreochromis niloticus). *Gene*, *687*, 109–115. doi:10.1016/j.gene.2018.11.016 |
| ***B. Foxl_2_ / Foxl_3_***  **FOXL_2_ (Forkhead transcription factor 2), member of the winged helix/ fork-head group of proteins known for ovarian differentiation** | | | | | |
| 2004 | | *Oncorhynchus mykiss* | | *Foxl_2_a* expression correlated with the level of aromatase. | Baron, D., Cocquet, J., Xia, X., Fellous, M., Guiguen, Y., & Veitia, R. A. (2004). An evolutionary and functional analysis of FoxL2 in rainbow trout gonad differentiation. *Journal of molecular endocrinology*, *33*(3), 705–715. doi:10.1677/jme.1.01566 |
| 2004 | *Oreochromis niloticus* | | | *Foxl_2_* is involved in fish gonad differentiation and the maintenance of ovarian function. | Wang, D., Kobayashi, T., Zhou, L., & Nagahama, Y. (2004). Molecular cloning and gene expression of Foxl2 in the Nile tilapia, Oreochromis niloticus. *Biochemical and biophysical research communications*, *320*(1), 83–89.  doi:10.1016/j.bbrc.2004.05.133 |
| 2006 | *Oryzias latipes* | | *Foxl_2_* was not involved in Ovarian determination, but was involved in differentiation of granulosa cells. | | Nakamoto, M., Matsuda, M., Wang, D. S., Nagahama, Y., & Shibata, N. (2006). Molecular cloning and analysis of gonadal expression of Foxl2 in the medaka, Oryzias latipes. *Biochemical and biophysical research communications*, *344*(1), 353–361. doi:10.1016/j.bbrc.2006.03.137 |
| 2007 | *Oreochromis niloticus* | | By regulating aromatase expression and possibly the entire steroidogenic pathway, *Foxl_2_* plays a decisive role in the ovarian differentiation. | | Wang, D. S., Kobayashi, T., Zhou, L. Y., Paul-Prasanth, B., Ijiri, S., Sakai, F., Okubo, K., Morohashi, K., & Nagahama, Y. (2007). Foxl2 up-regulates aromatase gene transcription in a female-specific manner by binding to the promoter as well as interacting with ad4 binding protein/steroidogenic factor 1. *Molecular endocrinology (Baltimore, Md.)*, *21*(3), 712–725. doi:10.1210/me.2006-0248 |
| 2007 | *Paralichthys olivaceus* | | FSH signaling and *Foxl_2_* are involved in the transcriptional regulation of *Cyp19a1*gene during gonadal sex differentiation in Japenese flounder with temperature dependent sex determination. | | Yamaguchi, T., Yamaguchi, S., Hirai, T., & Kitano, T. (2007). Follicle-stimulating hormone signaling and Foxl2 are involved in transcriptional regulation of aromatase gene during gonadal sex differentiation in Japanese flounder, Paralichthys olivaceus. *Biochemical and biophysical research communications*, *359*(4), 935–940.  doi:10.1016/j.bbrc.2007.05.208 |
| 2007 | *Scyliorhinus canicula* | | *Foxl_2_* is a marker for the mandibular mesoderm and gill buds and its expression is conserved in the perioptic mesenchyme, developing eyelids and pituitary. | | Wotton, K. R., French, K. E., & Shimeld, S. M. (2007). The developmental expression of foxl2 in the dogfish Scyliorhinus canicula. *Gene expression patterns : GEP*, *7*(7), 793–797.  doi:10.1016/j.modgep.2007.05.003 |
| 2008 | *Epinephelus merra* | | *Foxl_2_* downregulation most likely facilitates oocyte degeneration. | | Alam, M. A., Kobayashi, Y., Horiguchi, R., Hirai, T., & Nakamura, M. (2008). Molecular cloning and quantitative expression of sexually dimorphic markers Dmrt1 and Foxl2 during female-to-male sex change in Epinephelus merra. *General and comparative endocrinology*, *157*(1), 75–85. doi:10.1016/j.ygcen.2008.03.018 |
| 2008 | *Oreochromis niloticus* | | *Foxl_2_* and *Cyp19a1a* in XX gonads during early gonadal differentiation is critical for undifferentiated gonads to differentiate into the ovary in Nile tilapia. | | Ijiri, S., Kaneko, H., Kobayashi, T., Wang, D. S., Sakai, F., Paul-Prasanth, B., Nakamura, M., & Nagahama, Y. (2008). Sexual dimorphic expression of genes in gonads during early differentiation of a teleost fish, the Nile tilapia Oreochromis niloticus. *Biology of reproduction*, *78*(2), 333–341. doi:10.1095/biolreprod.107.064246 |
| 2010 | *Silurus meridionalis* | | *Dmrt1*, *Foxl_2_*, and *Cyp19a* were closely related to catfish sex differentiation, and the gonadotropin subunits were possibly related to ovarian differentiation and oocyte development. Estrogen was highly responsible for the ovarian differentiation and feminization of catfish fry under artificial propagation. | | Liu, Z. H., Zhang, Y. G., & Wang, D. S. (2010). Studies on feminization, sex determination, and differentiation of the Southern catfish, Silurus meridionalis--a review. *Fish physiology and biochemistry*, *36*(2), 223–235. doi:/10.1007/s10695-008-9281-7 |
| 2011 | *Clarias gariepinus* | | Sex-specific expression of *Cyp19a1* and *Foxl_2_* in females plays a critical role in ovarian development. | | Raghuveer, K., Senthilkumaran, B., Sudhakumari, C. C., Sridevi, P., Rajakumar, A., Singh, R., Murugananthkumar, R., & Majumdar, K. C. (2011). Dimorphic expression of various transcription factor and steroidogenic enzyme genes during gonadal ontogeny in the air-breathing catfish, Clarias gariepinus. *Sexual development : genetics, molecular biology, evolution, endocrinology, embryology, and pathology of sex determination and differentiation*, *5*(4), 213–223.  doi:10.1159/000328823 |
| 2011 | *Gobiocypris rarus* | | *Foxl_2_* plays an important role in female development. | | Jiang, W., Yang, Y., Zhao, D., Liu, X., Duan, J., Xie, S., & Zhao, H. (2011). Effects of sexual steroids on the expression of foxl2 in Gobiocypris rarus. *Comparative biochemistry and physiology. Part B, Biochemistry & molecular biology*, *160*(4), 187–193. doi:10.1016/j.cbpb.2011.08.005 |
| 2011 | *Clarias gariepinus* | | *Foxl_2_* is designated as a potential ovary and brain marker during female sex development. | | Sridevi, P., & Senthilkumaran, B. (2011). Cloning and differential expression of FOXL2 during ovarian development and recrudescence of the catfish, Clarias gariepinus. *General and comparative endocrinology*, *174*(3), 259–268. doi:10.1016/j.ygcen.2011.08.015 |
| 2013 | *Oreochromis niloticus* | | *Foxl_2_* and *Dmrt1* play antagonistic roles in sex differentiation via regulating *cyp19a1a* expression and estrogen production. | | Li, M. H., Yang, H. H., Li, M. R., Sun, Y. L., Jiang, X. L., Xie, Q. P., Wang, T. R., Shi, H. J., Sun, L. N., Zhou, L. Y., & Wang, D. S. (2013). Antagonistic roles of Dmrt1 and Foxl2 in sex differentiation via estrogen production in tilapia as demonstrated by TALENs. *Endocrinology*, *154*(12), 4814–4825. doi:10.1210/en.2013-1451 |
| 2013 | *Sebastes schlegeli* | | The expression pattern of *Foxl_2_* as well as aromatases may imply the role of *Foxl_2_* in the up-regulation of aromatases not only in the female fish but also in male. | | Mu, W. J., Wen, H. S., Li, J. F., & He, F. (2013). Cloning and expression analysis of Foxl2 during the reproductive cycle in Korean rockfish, Sebastes schlegeli. *Fish physiology and biochemistry*, *39*(6), 1419–1430.  doi:10.1007/s10695-013-9796-4 |
| 2014 | *Monopterus albus* | | For the development and maintenance of ovarian function, *Foxl_2_* plays a pivotal role. | | Hu, Q., Guo, W., Gao, Y., Tang, R., & Li, D. (2014). Molecular cloning and analysis of gonadal expression of Foxl2 in the rice-field eel Monopterus albus. *Scientific reports*, *4*, 6884.  doi:10.1038/srep06884 |
| 2015 | *Scatophagus argus* | | *Foxl_2_* plays an essential role in sexual development, and imply that it may regulate *Cyp19a1a* and *Cyp19a1b* expression. | | Liu, H., Mu, X., Gui, L., Su, M., Li, H., Zhang, G., Liu, Z., & Zhang, J. (2015). Characterization and gonadal expression of FOXL2 relative to Cyp19a genes in spotted scat Scatophagus argus. *Gene*, *561*(1), 6–14. doi:10.1016/j.gene.2014.12.060 |
| 2015 | *Channa argus* | | *Foxl_2_* may have basic function in granulosa cell differentiation and the maintenance of oocytes. | | Wang, D. D., Zhang, G. R., Wei, K. J., Ji, W., Gardner, J. P., Yang, R. B., & Chen, K. C. (2015). Molecular identification and expression of the Foxl2 gene during gonadal sex differentiation in northern snakehead Channa argus. *Fish physiology and biochemistry*, *41*(6), 1419–1433.  doi:10.1007/s10695-015-0096-z |
| 2015 | *Danio rerio* | | *Foxl_2_* plays a critical role in regulation of *Cyp19a1a.* | | Caulier, M., Brion, F., Chadili, E., Turies, C., Piccini, B., Porcher, J. M., Guiguen, Y., & Hinfray, N. (2015). Localization of steroidogenic enzymes and Foxl2a in the gonads of mature zebrafish (Danio rerio). *Comparative biochemistry and physiology. Part A, Molecular & integrative physiology*, *188*, 96–106. doi:10.1016/j.cbpa.2015.06.016 |
| 2015 | *Oryzias latipes* | | FOXL_3_ is a germ cell-intrinsic factor regulating gametogenic fate, sperms are produced in adult Folx_3_ null ovaries. | | Nishimura T, Sato T, Yamamoto Y, Watakabe I, et al. 2015. foxl3 is a germ cell-intrinsic factor involved in sperm-egg fate decision in medaka. Science 349: 328–31. doi: 10.1126/science.aaa2657 |
| 2017 | *Oreochromis niloticus* | | By upregulating *Cyp19a1a* expression and repressing male pathway gene expression, *Foxl_2_* promote ovarian development. | | Zhang, X., Li, M., Ma, H., Liu, X., Shi, H., Li, M., & Wang, D. (2017). Mutation of foxl2 or cyp19a1a Results in Female to Male Sex Reversal in XX Nile Tilapia. *Endocrinology*, *158*(8), 2634–2647. doi:10.1210/en.2017-00127 |
| 2017 | *Huso huso* | | *Foxl_2_* and *Cyp19a1a* mRNA expression is required for ovarian development. | | Yarmohammadi, M., Pourkazemi, M., & Kazemi, R. (2017). Differential expression of foxl2 and cyp19a1a mRNA during gonad developmental stages in great sturgeon Huso huso. *Journal of fish biology*, *90*(3), 1104–1111.  doi:10.1111/jfb.13224 |
| 2017 | *Danio rerio* | | *Foxl_2_* promotes ovarian development by upregulating *Cyp19a1a* expression and repressing male pathway gene expression. | | Zhang, X., Li, M., Ma, H., Liu, X., Shi, H., Li, M., & Wang, D. (2017). Mutation of foxl2 or cyp19a1a Results in Female to Male Sex Reversal in XX Nile Tilapia. *Endocrinology*, *158*(8), 2634–2647. doi:10.1210/en.2017-00127 |
| 2017 | *Danio rerio* | | *Foxl_2_a* and *Foxl_2_b* mutant show complete sex reversal and testis differentiation genes increases robustly. | | Yang, Y. J., Wang, Y., Li, Z., Zhou, L., & Gui, J. F. (2017). Sequential, divergent, and cooperative requirements of Foxl2a and Foxl2b in ovary development and maintenance of zebrafish. *Genetics*, *205*(4), 1551-1572.  doi: 10.1534/genetics.116.199133 |
| 2019 | *Paralichthys olivaceus* | | By maintaining *Cyp19a* expression and antagonizing the expression of Dmrt1, *Foxl2* may play a role in Ovarian differentiation. | | Fan, Z., Zou, Y., Liang, D., Tan, X., Jiao, S., Wu, Z., Li, J., Zhang, P., & You, F. (2019). Roles of forkhead box protein L2 (foxl2) during gonad differentiation and maintenance in a fish, the olive flounder (Paralichthys olivaceus). *Reproduction, fertility, and development*, *31*(11), 1742–1752.  doi:10.1071/RD18233 |
| 2020 | *Kryptolebias marmoratus* | | *Foxl_3_* seems to have an important role in oogenesis as well as spermatogenesis and gonadal structure. | | Qu, M., Ding, S., Schartl, M., & Adolfi, M. C. (2020). Spatial and temporal expression pattern of sex-related genes in ovo-testis of the self-fertilizing mangrove killifish (Kryptolebias marmoratus). *Gene*, *742*, 144581. doi:10.1016/j.gene.2020.144581 |
| C. ***Sox***  **Proteins encoded by Sox family of genes have a characteristic Sry related high mobility group (HMG) domain** | | | | | |
| 2003 | *Rice field eel* | | *Sox17* have potentially important roles in gonadal differentiation during sex reversal in this species. | | Wang, R., Cheng, H., Xia, L., Guo, Y., Huang, X., & Zhou, R. (2003). Molecular cloning and expression of Sox17 in gonads during sex reversal in the rice field eel, a teleost fish with a characteristic of natural sex transformation. *Biochemical and biophysical research communications*, *303*(2), 452–457.  doi:10.1016/s0006-291x(03)00361-9 |
| 2007 | *Epinephelus coioides* | | *Sox 3* has a potential regulatory role in oogenesis and germ cell differentiation. | | Yao, B., Zhou, L., Wang, Y., Xia, W., & Gui, J. F. (2007). Differential expression and dynamic changes of SOX3 during gametogenesis and sex reversal in protogynous hermaphroditic fish. *Journal of experimental zoology. Part A, Ecological genetics and physiology*, *307*(4), 207–219.  doi:10.1002/jez.361 |
| 2009 | *Acanthopagrus schlegeli* | | *Sox3* and *Dmrt1* are involved in the development of testis. | | Shin, H. S., An, K. W., Park, M. S., Jeong, M. H., & Choi, C. Y. (2009). Quantitative mRNA expression of sox3 and DMRT1 during sex reversal, and expression profiles after GnRHa administration in black porgy, Acanthopagrus schlegeli. *Comparative biochemistry and physiology. Part B, Biochemistry & molecular biology*, *154*(1), 150–156. doi:10.1016/j.cbpb.2009.05.013 |
| 2012 | *Dicentrarchus labrax.* | | *Sox19* is upregulated in the differentiation of ovary, but not in testis, suggesting a role in Ovarian differentiation. | | Navarro-Martín, L., Galay-Burgos, M., Piferrer, F., & Sweeney, G. (2012). Characterisation and expression during sex differentiation of Sox19 from the sea bass Dicentrarchus labrax. *Comparative biochemistry and physiology. Part B, Biochemistry & molecular biology*, *163*(3-4), 316–323. doi:10.1016/j.cbpb.2012.08.004 |
| 2014 | *Oryzias dancena.* | | Sex-reversed phenotypes in Sox3(Y) transgenic fish, and Sox3(Y) loss-of-function mutants all point to its critical role in sex determination. *Sox3* initiates testicular differentiation by upregulating expression of downstream *Gsdf,* which is highly conserved in fish sex differentiation pathways. | | Takehana, Y., Matsuda, M., Myosho, T., Suster, M. L., Kawakami, K., Shin-I, T., Kohara, Y., Kuroki, Y., Toyoda, A., Fujiyama, A., Hamaguchi, S., Sakaizumi, M., & Naruse, K. (2014). Co-option of Sox3 as the male-determining factor on the Y chromosome in the fish Oryzias dancena. *Nature communications*, *5*, 4157.  doi:10.1038/ncomms5157 |
| 2014 | *Clarias batrachus* | | These results suggest a possible role for *Sox3* has a possible role in the regulation of testicular development and function. | | Rajakumar, A., & Senthilkumaran, B. (2014). Expression analysis of sox3 during testicular development, recrudescence, and after hCG induction in catfish, Clarias batrachus. *Sexual development : genetics, molecular biology, evolution, endocrinology, embryology, and pathology of sex determination and differentiation*, *8*(6), 376–386. https://doi.org/10.1159/000368864 |
| 2015 | *Astyanax altiparanae* | | During the male reproductive cycle *Sox9* gets down-regulated after spermiation, indicating a role of these genes in spermatogenesis. | | Adolfi, M. C., Carreira, A. C., Jesus, L. W., Bogerd, J., Funes, R. M., Schartl, M., Sogayar, M. C., & Borella, M. I. (2015). Molecular cloning and expression analysis of dmrt1 and sox9 during gonad development and male reproductive cycle in the lambari fish, Astyanax altiparanae. *Reproductive biology and endocrinology : RB&E*, *13*, 2.  doi:10.1186/1477-7827-13-2 |
| 2016 | *Clarias batrachus* | | *Sox3* binds to the *11βhsd* gene and transactivates to regulate male  reproduction. | | Rajakumar, A., & Senthilkumaran, B. (2016). Sox3 binds to 11β-hydroxysteroid dehydrogenase gene promoter suggesting transcriptional interaction in catfish. *The Journal of steroid biochemistry and molecular biology*, *158*, 90–103. doi:10.1016/j.jsbmb.2016.01.003 |
| 2020 | *Cyprinus carpio* | | *Sox30* as well as *Sox9a/b* has influence in the regulation of testicular steroidogenesis. | | Anitha, A., & Senthilkumaran, B. (2020). Role of sox30 in regulating testicular steroidogenesis of common carp. *The Journal of steroid biochemistry and molecular biology*, *204*, 105769.  doi:10.1016/j.jsbmb.2020.105769 |
| **D.** ***Dax1***  **Dosage- sensitive sex reversal adrenal hypoplasia congenital critical region on the X chromosome gene, an orphan receptor is a member of the nuclear receptor superfamily (NR0B1).** | | | | | |
| 2007 | *Oryzias latipes* | | *Dax1* downegulates Ad4BP/Sf-1 and *Foxl_2_*-mediated P450arom expression in ovarias follicles. | | Nakamoto, M., Wang, D. S., Suzuki, A., Matsuda, M., Nagahama, Y., & Shibata, N. (2007). Dax1 suppresses P450arom expression in medaka ovarian follicles. *Molecular reproduction and development*, *74*(10), 1239–1246. doi:10.1002/mrd.20689 |
| 2015 | *Monopterus albus* | | The high level of *Amh* and a low level of *Dax1* expression are necessary for maintenance of testis function. | | Hu, Q., Guo, W., Gao, Y., Tang, R., & Li, D. (2015). Molecular cloning and characterization of amh and dax1 genes and their expression during sex inversion in rice-field eel Monopterus albus. *Scientific reports*, *5*, 16667. doi:10.1038/srep16667 |
| 2015 | *Danio rerio* | | *Dax1* is required for normal development and function of the interrenal organ. | | Zhao, Y., Yang, Z., Phelan, J. K., Wheeler, D. A., Lin, S., & McCabe, E. R. (2006). Zebrafish dax1 is required for development of the interrenal organ, the adrenal cortex equivalent. *Molecular endocrinology (Baltimore, Md.)*, *20*(11), 2630–2640.  doi:10.1210/me.2005-0445 |
| E. ***Ad4bp/Sf-1/ FtzF1***  **A fushi tarazu factor-1 (ftz-f1), a subfamily member of nuclear receptors is considered analogous to Sf-1 and certain reports include SF-1 as a member of ftz-f1.** | | | | | |
| 1999 | *Oryzias latipes* | | FTZ-F1 has a role in the transcriptional regulation of P-450arom in the ovarian follicle of medaka. | | Watanabe, M., Tanaka, M., Kobayashi, D., Yoshiura, Y., Oba, Y., & Nagahama, Y. (1999). Medaka (Oryzias latipes) FTZ-F1 potentially regulates the transcription of P-450 aromatase in ovarian follicles: cDNA cloning and functional characterization. *Molecular and cellular endocrinology*, *149*(1-2), 221–228.  doi:10.1016/s0303-7207(99)00006-4 |
| 2002 | *Salvelinus alpinus* | | FF1 is controlled by 17beta-estradiol. | | von Hofsten, J., Karlsson, J., Jones, I., & Olsson, P. E. (2002). Expression and regulation of fushi tarazu factor-1 and steroidogenic genes during reproduction in Arctic char (Salvelinus alpinus). *Biology of reproduction*, *67*(4), 1297–1304.  doi:10.1095/biolreprod67.4.1297 |
| 2003 | *Orechromis niloticus* | | *Ad4BP/Sf-1* probably acts as a transcriptional modulator to implement the paradoxical actions of gonadotropins on oP450arom gene. | | Yoshiura, Y., Senthilkumaran, B., Watanabe, M., Oba, Y., Kobayashi, T., & Nagahama, Y. (2003). Synergistic expression of Ad4BP/SF-1 and cytochrome P-450 aromatase (ovarian type) in the ovary of Nile tilapia, Oreochromis niloticus, during vitellogenesis suggests transcriptional interaction. *Biology of reproduction*, *68*(5), 1545–1553. doi:10.1095/biolreprod.102.010843 |
| 2007 | *Oreochromis niloticus* | | FOXL_2_ interact through the forkhead domain with the ligand-binding domain of *Ad4BP/Sf-1* to form a heterodimer and enhance the *Ad4BP/Sf-1* mediated *Cyp19a1* transcription. | | Wang, D. S., Kobayashi, T., Zhou, L. Y., Paul-Prasanth, B., Ijiri, S., Sakai, F., Okubo, K., Morohashi, K., & Nagahama, Y. (2007). Foxl2 up-regulates aromatase gene transcription in a female-specific manner by binding to the promoter as well as interacting with ad4 binding protein/steroidogenic factor 1. *Molecular endocrinology (Baltimore, Md.)*, *21*(3), 712–725.  doi:10.1210/me.2006-0248 |
| 2010 | *Cyprinus carpio* | | Involvement of SF-1 and *Cyp19a1b* in testis development and of *Cyp19a1a* in ovary development. | | Tang, B., Hu, W., Hao, J., & Zhu, Z. (2010). Developmental expression of steroidogenic factor-1, cyp19a1a and cyp19a1b from common carp (Cyprinus carpio). *General and comparative endocrinology*, *167*(3), 408–416. doi:10.1016/j.ygcen.2010.03.017 |
| 2011 | *Clarias gariepinus* | | Brain FTZ-F1 has potential roles in ovarian differentiation and recrudescence process probably through regulation of *Cyp19a1b* in teleosts. | | Sridevi, P., Dutta-Gupta, A., & Senthilkumaran, B. (2011). Molecular cloning and expression analysis of fushi tarazu factor 1 in the brain of air-breathing catfish, Clarias gariepinus. *PloS one*, *6*(12), e28867. doi:10.1371/journal.pone.0028867 |
| 2012 | *Oryzias latipes* | | During testicular development, *Hsd3b*, *Star* and *Ad4BP/Sf-1* were co-expressed in the interstitial somatic cells subsequent to the formation of the seminiferous tubule precursor, suggesting that *Ad4BP/Sf-1* regulated the transcription of both *Hsd3b* and *Star*. | | Nakamoto, M., Fukasawa, M., Tanaka, S., Shimamori, K., Suzuki, A., Matsuda, M., Kobayashi, T., Nagahama, Y., & Shibata, N. (2012). Expression of 3β-hydroxysteroid dehydrogenase (hsd3b), star and ad4bp/sf-1 during gonadal development in medaka (Oryzias latipes). *General and comparative endocrinology*, *176*(2), 222–230. doi:10.1016/j.ygcen.2012.01.019 |
| 2012 | *Oreochromis niloticus* | | FTZ-F1 belonged to Ad4BP SF-1/ group and FTZ-F1 transcripts were only expressed in the gonads and kidney but not in other tissues which specified role in gonadal development | | Cao, J., Chen, J., Jiang, Z., Luo, Y., & Gan, X. (2012). Molecular cloning and expression analysis of FTZ-F1 in the GIFT tilapia, Oreochromis niloticus. *Dong wu xue yan jiu = Zoological research*, *33*(E3-4), E40–E46. doi:10.3724/SP.J.1141.2012.E03-04E40 |
| 2012 | *Clarias gariepinus* | | FTZ-F1 and FOXL_2_ is involved in  transcriptional regulation of *Cyp19a1b* by in a teleost fish. | | Sridevi, P., Chaitanya, R. K., Dutta-Gupta, A., & Senthilkumaran, B. (2012). FTZ-F1 and FOXL2 up-regulate catfish brain aromatase gene transcription by specific binding to the promoter motifs. *Biochimica et biophysica acta*, *1819*(1), 57–66.  doi:10.1016/j.bbagrm.2011.10.003 |
| 2013 | *Sebastes schlegelii* | | The highest abundance of gene transcript was always observed in gonads of both juvenile and mature fish. In addition, the abundance of gene transcript in male tissues were higher than that in female tissue counterparts | | Shafi, M., Wang, Y., Zhou, X., Ma, L., Muhammad, F., Qi, J., & Zhang, Q. (2013). Isolation and expression analysis of FTZ-F1 encoding gene of black rock fish (Sebastes schlegelii). *Journal of Ocean University of China*, *12*(1), 183-189.  doi:[10.1007/s11802-013-1953-y](https://ui.adsabs.harvard.edu/link_gateway/2013JOUC...12..183S/doi:10.1007/s11802-013-1953-y) |
| 2016 | *Labeo rohita* | | HCG significantly induced expression of *Sf-1* gene and activation of SF-1 protein suggesting a link between SF-1 and P450 aromatase activation in fish ovary during gonadotropin-induced steroidogenesis. | | Roy Moulik, S., Pal, P., Majumder, S., Mallick, B., Gupta, S., Guha, P., Roy, S., & Mukherjee, D. (2016). Gonadotropin and sf-1 regulation of cyp19a1a gene and aromatase activity during oocyte development in the rohu, L. rohita. *Comparative biochemistry and physiology. Part A, Molecular & integrative physiology*, *196*, 1–10. doi:10.1016/j.cbpa.2016.02.004 |
| 2016 | *Oreochromis niloticus* | | *Sf-1* is a major regulator of steroidogenesis and reproduction in fish. SF-1 deficiency resulted in gonadal dysgenesis and feminization of XY gonads. | | Xie, Q. P., He, X., Sui, Y. N., Chen, L. L., Sun, L. N., & Wang, D. S. (2016). Haploinsufficiency of SF-1 Causes Female to Male Sex Reversal in Nile Tilapia, Oreochromis niloticus. *Endocrinology*, *157*(6), 2500–2514. doi:10.1210/en.2015-2049 |
| 2018 | *Trichogaster fasciata.* | | Localizations of aromatase and *Sf-1* in the cellular layer of oocytes demonstrated that aromatase is FSH-dependent and *Sf-1* could be regulated by both FSH and LH as relatively higher amount of aromatase was localized in the vitellogenic stage oocytes than the postvitellogenic and post germinal vesicle breakdown (post-GVBD) stages; whereas, high amount of SF-1 was observed in vitellogenic, postvitellogenic and post-GVBD stages. | | Guchhait, R., Chatterjee, A., Mukherjee, D., & Pramanick, K. (2018). Seasonal ovarian development in relation to the gonadotropins, steroids, aromatase and steroidogenic factor 1 (SF-1) in the banded gourami, Trichogaster fasciata. *General and comparative endocrinology*, *268*, 40–49. doi:10.1016/j.ygcen.2018.07.014 |
| F. ***CREB***  ***cAMP response element binding (Creb) protein*** | | | | | |
| 2009 | *Red spotted grouper* | | CREB regulation region from -1010 to -898 might be a major cis-acting element to Cyp19a1a promoter. | | Huang, W., Zhou, L., Li, Z., & Gui, J. F. (2009). Expression pattern, cellular localization and promoter activity analysis of ovarian aromatase (Cyp19a1a) in protogynous hermaphrodite red-spotted grouper. *Molecular and cellular endocrinology*, *307*(1-2), 224–236. doi:10.1016/j.mce.2009.04.003 |
| 2015 | *Oreochromis niloticus,*  *Catfish* | | Multiple creb like *Creb 1, Creb 2, Creb* 3 were found in Nile tilapia*.* CREB1 is likely to control oocyte growth, whereas CREB 2 and 3 contribute to oocyte maturation in tilapia  In catfish, a single form of CREB showed a maximum expression during spawning phase and hCG-induced maturation both in vivo and in vitro augmented CREB expression. | | Senthilkumaran, B., Sreenivasulu, G., Wang, D. S., Sudhakumari, C. C., Kobayashi, T., & Nagahama, Y. (2015). Expression Patterns of CREBs in Oocyte Growth and Maturation of Fish. *PloS one*, *10*(12), e0145182. doi:10.1371/journal.pone.0145182 |
