# Supplementary material for: Sex Specific Transcriptional Regulation of Gonadal Steroidogenesis in Teleost Fishes
Source: Front Endocrinol (Lausanne). 2022 Feb 18;13:820241. doi: 10.3389/fendo.2022.820241 (PMC8894591; doi:10.3389/fendo.2022.820241)
Supplement: Supplementary file 2 [file Table_2.docx]

**Supplementary Table 2 summarizes the effect of various environmental parameters on fish gonadal development and steroidogenesis.**

| **Parameters** | **Fish Species** | **Impact** | **References** |
| --- | --- | --- | --- |
| **Temperature** | *Pagrus major* | High water temperature suppressed both expression of P450arom and 11β-hydroxylase and the development of oocytes in ovarian portion of hermaphroditic gonads. | Lim, B. S., Kagawa, H., Gen, K., & Okuzawa, K. J. F. P. (2003). Effects of water temperature on the gonadal development and expression of steroidogenic enzymes in the gonad of juvenile red seabream, Pagrus major. *Fish Physiology and Biochemistry*, *28*(1), 161-162.  doi:10.1023/b:fish.0000030511.73742.35 |
|  | *Sander lucioperca* | Moderate temperatures (12^0^–15 °C for) for at least 3 months were required to proceed with first maturation in juvenile pikeperch. The most efficient effect was observed at 12 °C, while high (23 °C) or low (6 °C) temperatures prevented gonadal maturation. | Hermelink, B., Wuertz, S., Trubiroha, A., Rennert, B., Kloas, W., & Schulz, C. (2011). Influence of temperature on puberty and maturation of pikeperch, Sander lucioperca. *General and comparative endocrinology*, *172*(2), 282–292. doi:10.1016/j.ygcen.2011.03.013 |
|  | *Cottus gobio* | A 4°C rise in water temperature affects gametogenesis by advancing the spawning, and a complete reproductive failure is observed at an elevated temperature of 8°C. | Dorts, J., Grenouillet, G., Douxfils, J., Mandiki, S. N., Milla, S., Silvestre, F., & Kestemont, P. (2012). Evidence that elevated water temperature affects the reproductive physiology of the European bullhead Cottus gobio. *Fish physiology and biochemistry*, *38*(2), 389–399.  doi:10.1007/s10695-011-9515-y |
|  | *Periophthalmus modestus* | Vitellogenesis and spermatogenesis were induced by high temperature treatment (30 °C).Gonadal development did not occur in the low temperature treatment (18 °C). | Shiota, T., Ishimatsu, A., & Soyano, K. (2003). Effects of temperature on gonadal development of mudskipper (Periophthalmus modestus). *Fish Physiology and Biochemistry*, *28*(1), 445-446.  doi:10.1023/B:FISH.0000030624.35937.3d |
|  | *Kryptolebias marmoratus* | Elevated water temperature due to aquatic climate change prior to sexual maturation and the onset of spawning can lead to the reproductive dysfunction of hermaphroditic *K. marmoratus*. | Park, C. B., Kim, Y. J., & Soyano, K. (2017). Effects of increasing temperature due to aquatic climate change on the self-fertility and the sexual development of the hermaphrodite fish, Kryptolebias marmoratus. *Environmental science and pollution research international*, *24*(2), 1484–1494. doi:10.1007/s11356-016-7878-4 |
|  | *Scatophagus argus* | From 23^0^ to 29° C, the optimal temperature for ovarian development in *S. argus* was 23-26° C, and 6% fish oil supplementation could effectively promote ovarian development. Optimal temperature and fish oil supplement might increase ovarian Foxl_2_ mRNA expressions to promote ovarian development in *S. argus.* | Li, G. L., Zhang, M. Z., Deng, S. P., Chen, H. P., & Zhu, C. H. (2015). Effects of temperature and fish oil supplementation on ovarian development and foxl2 mRNA expression in spotted scat Scatophagus argus. *Journal of fish biology*, *86*(1), 248–260. doi:10.1111/jfb.12578 |
|  | Clarias batrachus | Growth Hormone influences the somatic growth and testicular activities depending on the temperature of the rearing water; warmer temperature and longer photoperiod promote testicular steroidogenic and spermatogenic activities in fish. | Gopal, R. N., Kumar, P., & Lal, B. (2014). Temperature dependent action of growth hormone on somatic growth and testicular activities of the catfish, Clarias batrachus. *General and comparative endocrinology*, *195*, 125-131.  doi:10.1016/j.ygcen.2013.11.003 |
|  | Gold fish | Genotypic females can be changed to phenotypic males by high temperature treatment. high temperature must be maintained during the Temperature Sensitive Period in order to produce a high proportion of masculinized genetic female goldfish. | Goto-Kazeto, R., Abe, Y., Masai, K., Yamaha, E., Adachi, S., & Yamauchi, K. (2006). Temperature-dependent sex differentiation in goldfish: establishing the temperature-sensitive period and effect of constant and fluctuating water temperatures. *Aquaculture*, *254*(1-4), 617-624.  doi:10.1016/j.aquaculture.2005.10.009 |
|  | Oreochromis niloticus | Temperature action on tilapia testis development induces the rapid increase of both *Dmrt1* and amh expressions followed by the down-regulation of *Foxl2* and *Cyp19a1a*. This suggests that *Dmrt1* and/or *Amh* may be the modulator(s) of the down-regulation of *Foxl2* and/or *Cyp19a1a*. | Poonlaphdecha, S., Pepey, E., Canonne, M., de Verdal, H., Baroiller, J. F., & D'Cotta, H. (2013). Temperature induced-masculinisation in the Nile tilapia causes rapid up-regulation of both dmrt1 and amh expressions. *General and comparative endocrinology*, *193*, 234–242.  doi:10.1016/j.ygcen.2013.06.007 |
|  | *Lepomis macrochirus* | The water temperature regulates the sex differentiation of bluegill through modulation of the expression of *Foxl2* and *Cyp19a1a* | Shen, Z. G., Eissa, N., Yao, H., Xie, Z. G., & Wang, H. P. (2018). Effects of Temperature on the Expression of Two Ovarian Differentiation-Related Genes *foxl2* and *cyp19a1a*. *Frontiers in physiology*, *9*, 1208. doi:10.3389/fphys.2018.01208 |
|  | European sea bass | In high temperature group, dmrt1, *Star*(steroidogenic acute regulatory protein), *nc3c1* ( glucocorticoid receptor) were upregulated whereas in low temperature group genes such as aromatase (*Cyp19a1a*), aquaporin 1 were downregulated. | Díaz, N., & Piferrer, F. (2015). Lasting effects of early exposure to temperature on the gonadal transcriptome at the time of sex differentiation in the European sea bass, a fish with mixed genetic and environmental sex determination. *BMC genomics*, *16*(1), 1-16.  doi:10.1186/s12864-015-1862-0 |
|  | Pejerrey | 11-Keto-testosterone detected in high levels in pejerrey larvae reared at high masculinizing temperature. | Fernandino, J. I., Hattori, R. S., Acosta, O. D. M., Strüssmann, C. A., & Somoza, G. M. (2013). Environmental stress-induced testis differentiation: androgen as a by-product of cortisol inactivation. *General and Comparative Endocrinology*, *192*, 36-44.  doi:10.1016/j.ygcen.2013.05.024 |
|  | *Oreochromis mossambicus* | Exposure to the lower temperature before 10 days old induced a high proportion of females whereas a high proportion of males was induced by the elevated temperature after 10 days old . Both lower and elevated temperatures induce the gonadal feminization and masculinization, respectively, during its restricted developmental period. | Wang, L. H., & Tsai, C. L. (2000). Effects of temperature on the deformity and sex differentiation of tilapia, Oreochromis mossambicus. *The Journal of experimental zoology*, *286*(5), 534–537.  doi:10.1002/(SICI)1097-010X(20000401)286:5%3C534::AID-JEZ11%3E3.0.CO;2-2 |
|  | *Odontesthes argentinensis* | The sex-ratios of *O. argentinensis* reared at 18^0^ or 21 °C were female-biased whereas those at 25 °C were not; groups reared at 18 °C had significantly more females than groups from the same progeny reared at 25 °C | Strüssmann, C. A., Cota, J. C. C., Phonlor, G., Higuchi, H., & Takashima, F. (1996). Temperature effects on sex differentiation of two South American atherinids, Odontesthes argentinensis and Patagonina hatcheri. *Environmental Biology of Fishes*, *47*(2), 143-154.  doi:10.1007/BF00005037 |
|  | *Dicentrarchus labrax* | Temperature during the very early developmental stages is the crucial factor affecting the process of sex differentiation of the sea bass, with low rearing temperatures (13^0^ or 15^0^ C) resulting in sex proportions consistently skewed in favor of females. | Pavlidis, M., Koumoundouros, G., Sterioti, A., Somarakis, S., Divanach, P., & Kentouri, M. (2000). Evidence of temperature-dependent sex determination in the European sea bass (Dicentrarchus labrax L.). *The Journal of experimental zoology*, *287*(3), 225–232.  doi:10.1002/1097-010x(20000801)287:3<225::aid-jez4>3.0.co;2-d |
|  | *Misgurnus anguillicaudatus* | High water temperature influences the sex of genetic females in the  loath and then induces sex reversal to physiological males | Nomura, T., Arai, K., Hayashi, T., & Suzuki, R. (1998). Effect of temperature on sex rations of normal and gynogenetic diploid loach. *Fisheries science*, *64*(5), 753-758.  doi:10.2331/fishsci.64.753 |
|  | *Oreochromis aureus* | High temperature regimes produced high male ratios (mean male percentage: 97.8%) while intermediate thermal regime gave balanced ratios (mean male percentage: 63.0%). Low temperature delayed the differentiation of gonads | Desprez, D., & Mélard, C. (1998). Effect of ambient water temperature on sex determinism in the blue tilapia Oreochromis aureus. *Aquaculture*, *162*(1-2), 79-84.  doi:10.1016/S0044-8486(97)00242-1 |
| **Hypoxia** | Zebra fish | In zebrafish, hypoxia has been shown to down‐regulate *Cyp19* and alter the ratio of testosterone to estradiol during early sex development, leading to a male‐biased F1 generation. | Wu, R. S. (2009). Effects of hypoxia on fish reproduction and development. In *Fish physiology* (Vol. 27, pp. 79-141). Academic Press.  doi:10.1016/S1546-5098(08)00003-4 |
| **Photoperiod** | *Salvelinus fontinalis* | The short-term expansion of the photo period delayed maturation and increased the growth rate of brook trout. | Lundova, K., Matousek, J., Prokesova, M., Sebesta, R., Policar, T., & Stejskal, V. (2019). The effect of timing of extended photoperiod on growth and maturity of brook trout (Salvelinus fontinalis). *Aquaculture Research*, *50*(6), 1697-1704  doi:10.1111/are.14053. |
|  | *Oreochromis niloticus* L. | A 12L:12D photoperiod regime should be adopted for maximum fecundity, seed production and spawning frequencies of Nile tilapia broodstock reared in intensive, recirculating systems. If maximum reproduction is desired, a near-natural day length photoperiod should be used. | El‐Sayed, A. F. M., & Kawanna, M. (2007). Effects of photoperiod on growth and spawning efficiency of Nile tilapia (Oreochromis niloticus L.) broodstock in a recycling system. *Aquaculture Research*, *38*(12), 1242-1247.  doi:10.1111/j.1365-2109.2007.01690.x |
|  | Danio rerio | The outcome indicates that extending day length affects both growth and spawning of zebrafish. Together, these data suggest that a photoperiod of 16L:08D might be optimal for laboratory zebrafish, at least from a production standpoint. | Abdollahpour, H., Falahatkar, B., & Lawrence, C. (2020). The effect of photoperiod on growth and spawning performance of zebrafish, Danio rerio. *Aquaculture Reports*, *17*, 100295.  doi:10.1016/j.aqrep.2020.100295 |
|  | Dicentrarchus labrax*, L.* | The present results indicate a significant beneficial effect of constant long and expanded photoperiods on reducing sea bass gonadal development and enhancing growth at the age of [commercialisation](https://www.sciencedirect.com/topics/agricultural-and-biological-sciences/commercialization), with a potential application in [aquaculture](https://www.sciencedirect.com/topics/agricultural-and-biological-sciences/aquaculture). | Rodrı́guez, L., Zanuy, S., & Carrillo, M. (2001). Influence of daylength on the age at first maturity and somatic growth in male sea bass (Dicentrarchus labrax, L.). *Aquaculture*, *196*(1-2), 159-175.  doi:10.1016/S0044-8486(00)00555-X |
|  | *Pseudorasbora parva* | The growth of juvenile *P. parva* can be stimulated significantly by constant–long photoperiods and that these photoperiods can advance sexual maturity of females by approximately 2 months. | Zhu, D., Yang, K., Gul, Y., Song, W., Zhang, X., & Wang, W. (2014). Effect of photoperiod on growth and gonadal development of juvenile Topmouth Gudgeon Pseudorasbora parva. *Environmental biology of fishes*, *97*(2), 147-156.  doi:10.1007/s10641-013-0133-7 |
|  | *Mugil cephalus* | Short photoperiod (06L:18D) along with the ambient temperature (21 ^0^C) was found to be effective in stimulating vitellogenesis in grey mullet, *Mugil cephalus* thus triggering ovarian recrudescence independent of season | Kuo, C. M., & Nash, C. E. (1975). Recent progress on the control of ovarian development and induced spawning of the grey mullet (Mugil cephalus L.). *Aquaculture*, *5*(1), 19-29.  doi:10.1016/0044-8486(75)90014-9 |
| **Dietary habit** | Pelteobagrus fulvidraco | The level of serum testosterone decreased with increasing diet ratios of Linolenic acid (LNA) to Linoleic acid (LA). The mRNA levels of steroidogenesis related genes(*Cyp19a*, *Cyp17a1,erβ*) were significantly downregulated with increasing dietary LNA/LA ratios in Ovary of female. LH,FSH and mRNA level of *20β-hsd* upregulated with increasing LNA/LA ratios | Fei, S., Liu, C., Xia, Y., Liu, H., Han, D., Jin, J., ... & Xie, S. (2020). The effects of dietary linolenic acid to linoleic acid ratio on growth performance, tissues fatty acid profile and sex steroid hormone synthesis of yellow catfish Pelteobagrus fulvidraco. *Aquaculture Reports*, *17*, 100361.  doi:10.1016/j.aqrep.2020.100361 |
|  | Gold fish | AA formed subsequent to the action of phospholipase C on membrane phospholipids has a role in the regulation of steroidogenesis in preovulatory goldfish ovarian follicles. | Van der Kraak, G., & Chang, J. P. (1990). Arachidonic acid stimulates steroidogenesis in goldfish preovulatory ovarian follicles. *General and comparative endocrinology*, *77*(2), 221–228.  doi:10.1016/0016-6480(90)90306-7 |
|  | *Gadus morhua* | Plasma concentrations of vitellogenin, estradiol-17β and testosterone and key gene transcript levels were affected by dietary ARA and stage of maturation. The results show that ARA has a significant influence on the reproductive physiology of female Atlantic cod. | Norberg, B., Kleppe, L., Andersson, E., Thorsen, A., Rosenlund, G., & Hamre, K. (2017). Effects of dietary arachidonic acid on the reproductive physiology of female Atlantic cod (Gadus morhua L.). *General and comparative endocrinology*, *250*, 21–35.  doi:10.1016/j.ygcen.2017.05.020 |
|  | *Cynoglossus semilaevis* | Results showed that ARA supplementation significantly reduced the estradiol production in females, but stimulated the testosterone production in males. ARA supplementation significantly reduced the mRNA expression of aromatase in ovaries but significantly increased the gene expression of 3β-hydroxysteroid dehydrogenase (*3β-Hsd*) in testes. | Xu, H., Cao, L., Zhang, Y., Johnson, R. B., Wei, Y., Zheng, K., & Liang, M. (2017). Dietary arachidonic acid differentially regulates the gonadal steroidogenesis in the marine teleost, tongue sole (Cynoglossus semilaevis), depending on fish gender and maturation stage. *Aquaculture*, *468*, 378-385.  doi:10.1016/j.aquaculture.2016.11.002 |
|  | *Cynoglossus semilaevis* | Females seemed to require more EPA but less DHA for the gonadal steroidogenesis than males. | Xu, H., Cao, L., Wei, Y., Zhang, Y., & Liang, M. (2017). Effects of different dietary DHA:EPA ratios on gonadal steroidogenesis in the marine teleost, tongue sole (Cynoglossus semilaevis). *The British journal of nutrition*, *118*(3), 179–188. doi:10.1017/S0007114517001891 |
| **pH** | *Salvelinus fontinalis* | Females avoided upwellings of pH 4.0 and 4.5 in preference to alkaline and ambient upwelling | Johnson, D. W., & Webster, D. A. (1977). Avoidance of low pH in selection of spawning sites by brook trout (Salvelinus fontinalis). *Journal of the Fisheries Board of Canada*, *34*(11), 2210-2215.  doi:10.1139/f77-293 |
|  | *Brook trout* | The disruptive physiological changes and (or) reduced nutritional status associated with acid stress are responsible for the reduction in activity of the gonadotropes and oocyte atresia. | Tam, W. H., Fryer, J. N., Valentine, B., & Roy, R. J. J. (1990). Reduction in oocyte production and gonadotrope activity, and plasma levels of estrogens and vitellogenin, in brook trout exposed to low environmental pH. *Canadian journal of zoology*, *68*(12), 2468-2476.  **doi:**10.1139/z90-345 |
|  | *Coregonus wartmanni* | Ovulation was delayed at pH 4.75 with aluminium and without aluminium and Regression of testis was delayed at pH 4.75 and most prominently at pH 4.75 with aluminium. | Vuorinen, P. J., Vuorinen, M., & Peuranen, S. (1990). Long-term exposure of adult whitefish (Coregonus wartmanni) to low pH/aluminium: effects on reproduction, growth, blood composition and gills. *Acidification in Finland, Berlin, Springer-Verlag*, 941-961.  doi:10.1007/978-3-642-75450-0_46 |
|  | *Salvelinus fontinalis* | Initiation of gametogenesis was not affected over the range of pH. Ovulation was significantly delayed in the acidic groups. | Tam, W. H., & Payson, P. D. (1986). Effects of chronic exposure to sublethal pH on growth, egg production, and ovulation in brook trout, Salvelinus fontinalis. *Canadian Journal of Fisheries and Aquatic Sciences*, *43*(2), 275-280.  doi:10.1139/f86-035 |
|  | *Clarias gariepinus* | Reduced pH condition has a depressing effect on the growth rate of *Clarias gariepinus* fry | Ndubuisi, U. C., Chimezie, A. J., Chinedu, U. C., Chikwem, I. C., & Alexander, U. (2015). Effect of pH on the growth performance and survival rate of Clarias gariepinus fry. *International Journal of Research in Biosciences*, *4*(3), 14-20. |
| **Salinity** | *Psammoperca waigiensis* | There is a salinity limits for oocyte maturation or ovulation and subsequent reduction in spawning, but embryonic development requires a higher salinity. The effect of salinity on embryo development and hatching rate suggests that in breeding season the brood fish spawned in areas where the salinity and other parameters are favorable. | Pham, H. Q., Kjørsvik, E., Nguyen, A. T., Nguyen, M. D., & Arukwe, A. (2010). Reproductive cycle in female Waigieu seaperch (Psammoperca waigiensis) reared under different salinity levels and the effects of dopamine antagonist on steroid hormone levels. *Journal of Experimental Marine Biology and Ecology*, *383*(2), 137-145.  doi:10.1016/j.jembe.2009.12.010 |
|  | *Eriocheir sinensis* | The elevating salinity promote gonadal development, increase hemolymph osmolality and K^+^ and Mg^+^ concentrations. The brackish water promote gonadal development of male and increase osmolality and ionic concentration in hemolymph while reduced activity of Na^+^/K ^+^–ATPase and its mRNA expression in the posterior gills as well as metabolism | Long, X., Wu, X., Zhao, L., Ye, H., Cheng, Y., & Zeng, C. (2017). Effects of salinity on gonadal development, osmoregulation and metabolism of adult male Chinese mitten crab, Eriocheir sinensis. *PLoS One*, *12*(6), e0179036.  doi:10.1371/journal.pone.0179036 |
|  | *Oreochromis niloticus* | Gonadal development and onset of reproduction can be delayed by high salinities, | Fineman‐Kalio, A. S. (1988). Preliminary observations on the effect of salinity on the reproduction and growth of freshwater Nile tilapia, Oreochromis niloticus (L.), cultured in brackishwater ponds. *Aquaculture Research*, *19*(3), 313-320.  doi:10.1111/j.1365-2109.1988.tb00435.x |
|  | *Acanthopagrus butcheri* | Plasma estradiol (E_2_) and testosterone (T) levels in saline-injected fish, either remained low or were significantly suppressed. | Haddy, J. A., & Pankhurst, N. W. (2000). The effects of salinity on reproductive development, plasma steroid levels, fertilisation and egg survival in black bream Acanthopagrus butcheri. *Aquaculture*, *188*(1-2), 115-131.  doi:10.1016/S0044-8486(00)00326-4 |
|  | *Diplodus vulgaris* | Exposure to salinity caused the deleterious effect on the gonads. Hypertrophy followed by degeneration of spermatogonia and deformation of seminiferous lobules, cessation of cell division and deformation of the oocytes were observed. | Moharram, S. (2000). EFFECT OF SALINITY ON GONAD DEVELOPMENT IN DIPLODUS VULGAR1S (FAMILY: SPARIDAE) DURING THE BREEDING SEASON. *Egyptian Journal of Aquatic Biology and Fisheries*, *4*(1), 139-160.  doi:10.21608/ejabf.2000.1645 |
| **Population density** | *Poecilia reticulata* | After one gestation, females at the lowest population level had greater ovarian length, height, gonad weight, width and volume compared with those from the higher density. The average gonadosomatic ratio was highest in the lowest population density | Dahlgren, B. T. (1979). The effects of population density on fecundity and fertility in the guppy, Poecilia reticulata (Peters). *Journal of Fish Biology*, *15*(1), 71-91.  doi:10.1111/j.1095-8649.1979.tb03573.x |
